# Japanese sound-symbolic words in global contexts: from translation to hybridization

**DOI:** 10.12688/f1000research.55546.1

**Published:** 2021-10-08

**Authors:** Noriko Hiraishi

**Affiliations:** 1 Faculty of Humanities and Social Sciences, University of Tsukuba, Tsukuba, Ibaraki, 3058571, Japan

**Keywords:** Sound-symbolic words, Modern Japanese poetry, Manga (Japanese comics), Translation, Hybridization, "Third Space"

## Abstract

This paper explores the global reception and development of the artistic expression of onomatopoeia and mimetic words in modern and contemporary Japanese literary texts adopting the method of comparative literature. By analyzing sound-symbolic words and their translations in modern Japanese poetry and contemporary comics, the intercultural dialogues of these texts are examined and the emergence of hybrid onomatopoeia in global comic works is illuminated. The Japanese language is often noted for its richness of sound-symbolic words. In the literary world, modern poetry adopted and elaborated the use of these words from the late 19th century in its quest for a new style of poetry. In the early 20th century, poets developed the artistic expression of sound-symbolic words and succeeded in giving musicality to the “new-style poem”. However, the translation of Japanese sound-symbolic words has always been problematic. Experimental uses of these words in modern poems were often untranslatable, making the translations incomprehensible or dull. Nevertheless, graphic narratives and their worldwide distribution changed that situation. Japanese comics (manga) has particularly developed the artistic expression of sound-symbolic words. Usually placed outside speech balloons, these words are elaborately depicted and are important elements of the panel/page layout. Notably, the global popularity of the genre developed a new phase of intercultural dialogue. As not every word has an equivalent or is translatable in the target language, translators have left sound-symbolic words untouched in the translated versions, putting translation aside. Thus, the combination of Japanese and the target language seems to influence the visual comprehension of sound effects among the readers. Through the examinations of some cases, this paper brings to light the emergence of some hybrid onomatopoeia and reveals that the “Third Space” formed by the translation and hybridization of manga is a dynamic field that creates a new culture.

## Introduction

Onomatopoeia and mimetic words have always colored our languages and literature. The Japanese language is often noted for its richness of these sound-symbolic words, and has around 4,500 of them ( Ono, 2007). Sound-symbolic words are usually classified into three or five groups ( Iwasaki, 2013: 69; Kindaichi, 1978: 5–8; Shibatani, 2006: 154) as follows:

1. Phonomimes (onomatopoeia)
  ➢ Animate phonomime ( *giseigo*)
  voice-mimicking words: words that mimic sounds made by living things
  ➢ Inanimate phonomime ( *giongo*)
  sound-mimicking words: words that mimic sounds made by inanimate objects
2. Phenomimes (mimetic words)
  ➢ Animate phenomime ( *gitaigo*)
  manner-mimicking words for living things
  ➢ Inanimate phenomime ( *giyōgo*)
  condition-mimicking words for inanimate objects
3. Psychomimes
  ➢ Psychological/physiological-state-mimicking words ( *gijōgo*)

However, writers of the late twentieth century have not always appreciated this abundance of sound-symbolic words in the Japanese language. For instance, Yukio Mishima criticizes the use of onomatopoeia in fiction:

Onomatopoeia brings daily conversation to life and gives it expressive power, but at the same time it typifies expression and makes it vulgar. (...) You will still find an onomatopoeia of laughter in popular literature, such as “All right, ha-ha…,” but everyone would be aware of the childishness of this technique.
  ( Mishima, 1995: 140–141)

Another Japanese writer, Saiichi Maruya, takes a more neutral position, yet he also admits “the childishness” of sound-symbolic words ^1^:

Japanese language is abundant with these phonomimes and phenomimes. If you abuse them, you will give an impression of being childish, whereas it would be cold and hollow if you reject them strictly. ( Maruya, 1977: 221)

This paper explores the artistic expression of onomatopoeia and mimetic words in Japanese literary texts. Although the stigma that renowned writers have placed on the use of sound-symbolic words has influenced Japanese fiction in the 20 ^th^ century, ^2^ modern Japanese poetry has cultivated such use of sound-symbolic words with a completely different attitude. Moreover, by considering sound-symbolic words in contemporary comics, this paper also reveals the contribution of sound-symbolic words to intercultural dialogue.

Regarding Japanese comics in global contexts, the polysystem theory developed by Itamar Even-Zohar has contributed to deepening the debate on this issue ( Rampant, 2010; Sell, 2011). As for the sound-symbolic words transcending language and culture, the discussion of the “Third Space” in recent translation studies also has great implications. Homi Bhabha argues that translation, as “the performative aspect of cultural communication” ( Bhabha, 2004: 326), creates a “Third Space”, a boundary point where cultures collide and mix. Referring to Bhabha’s theories, especially those of “hybridity” and “in-between”, Michaela Wolf draws translation out of the bilingual problem into a different phase:

If we consider the *Third Space* as the potential and starting point for interventionist translation strategies, we realize that such strategies go far beyond the traditional concepts of “original” and “translation”, and the old dichotomy of “foreignizing” versus “domesticating” in all its implications. These strategies imply a shift toward the centre, where cultures encounter each other, and where meanings are effectively “remixed” (as shown in the example of *liget*). The place where cultures overlap and *hybridity* comes into being can already be considered as the locus of translation. This implies that culture is already itself translation. ( Wolf, 2000: 141)

From this perspective, translation “no longer bridges a gap between two different cultures but becomes a strategy of intervention through which newness comes into the world, where cultures are remixed” ( Simon, 2000: 21). Adopting this point of view, this paper examines the intercultural dialogues of modern Japanese poetry and contemporary comics analyzing sound-symbolic words in these texts through translation, and argues for the emergence of some hybrid onomatopoeias in global comic works.

This study adopts the method of comparative literature, aiming to strike a balance between descriptive and interpretive case study approaches. The former approach implies historical and empirical knowledge, while the latter seeks to develop conceptual categories through close reading of the texts and to interpret the data. The paper, which is a series of case studies, does not allow us to draw any corpus-based conclusions, but it does allow us to identify the characteristics of each case and how it acquires meaning in different contexts. The data is extracted from published books, not from the first version published in magazines or other sources, except for the case of Indonesia, where several comic magazines have been published as a forum for the publication of original works.

## “Poem” and “Song”: The quest for musicality in modern poetry

Let us briefly review the quest for a new style of poetry in modern Japan. Since Japan opened its ports to Western countries in the 1860s, the Japanese had been immensely impressed with and influenced by Western culture. Japan’s modernization was, as is often pointed out (e.g., Miyoshi, 1972), a cultural westernization. Art and literature played important roles in this process, sometimes prompting modifications and reconstructions of cultural memories, such can be seen in Japanese customs and the gap between the written and spoken languages. In the field of poetry, a movement to create a new form of Japanese poetry, abandoning the conventional Japanese formulas (5-7-5-7-7 and 5-7-5 syllable meters) and the Chinese-style, emerged under the influence of European literature. *Shintaishishō* (A Selection of Poems in the New Style), which sought to include some ideological and abstract content, incorporating stanzaic forms, rhymes and refrains, was published in August 1882. Translators who published translations of European poems also contributed to the development of shintaishi (new-style poem) as a new form of poetry.

It is worth noting that a fusion of poetry and western music was pursued in the process. Shūji Izawa introduced *Shōka,* a new word for song, to primary schools as an attempt to incorporate Western sounds into education. The three volumes of the *Shōgaku shōka shū* (Elementary School Shōka Collection) were published from 1881 to 1884, in which lyrics suitable to “cultivate virtue (Izawa)” ( Tokyo University of the Arts and Centennial History Editorial Committee, 1987: 116) were put to the melody of hymns.

First Be Fragrant

1. Be fragrant, fragrant. Cherry tree of the garden.
2. Stop, rest. Firefly on wild flowers.
3. Wave, bend. Eulalia in the field.
4. Cry, fly. Plover at the shallows. (lyrics by Chikai Inagaki)

As the first song of the collection’s first volume depicts the four seasons, its lyrics “incorporated plenty of poetic imagery of post- *Man’yōshū* (Collection of Ten Thousand Leaves) nature and human affairs in the Japanese islands” ( Haga, 2002: 28). The lyrics follow the 2/4 time of the melody: ka/o/re/e, ni/o/e/e, so/no/u/no, sa/ku/ra/a.

In 1894, Tomoki Owada evaluated *A Selection of Poems in the New Style* and *Elementary School Shōka Collection*, stating that the former “pioneered the development of new-style poetry” ( Santo, 2008: 150). He appreciated this work because it “tried to engender so-called *Poemu (poem)* in plain and simple words”, while the *Elementary School Shōka Collection* “was a model of so-called *Songu (song)* with lyrics often antiquated and old-fashioned”. Soon after this evaluation, *shōka* began to function as a device for “national education”. Bimyō Yamada was a poet who devoted his life to create *shōka* in the movement to unify the written and spoken styles of Japanese. His most popular *shōka* turned out to be a military song called *Teki ha ikuman* (Tens of Thousands of Enemies, 1891), with the melody composed by a professor at Tokyo Music Academy. Meanwhile, *Omokage* (Vestiges, 1889), an anthology of translated poems by Ogai Mori, among others, greatly influenced the literary world. The literati who absorbed Western culture through this anthology soon began to consider “music and poetry as an art” and tried to pursue the musicality of modern poetry. In a letter to his brother on November 29, 1894, Chogyū Takayama shared that in a concert he attended at Ueno Music Academy, where “piano, violin, *shōka*, military songs, sword dance, etc.” were played and performed, he was impressed with an art song called *Autumn Breeze* with lyrics by Bimyō Yamada:

Until today I thought that the *shōka* was a boring thing, but when I heard this, I realized that *shōka* could be a refined and elegant genre compared to Japanese music. ( Takayama, 1933: 58)

It seems that his preference toward “refined and elegant” songs, which meant songs composed to good poems, influenced the literary world. We can see the continuance of this inclination in the 20 ^th^ century, in a popular novel serialized in *Yomiuri Newspaper* in 1905: Fūyo Oguri’s *Seishun* (Youth). The novel starts with a recitation of a new-style poem, written by the main character, Kin’ya Seki. Praised highly by his friends, the poem was put to music and performed in a concert, which became a great success. Through the hero of this popular work of fiction depicted as a “new intellectual man”, we see that new-style poets at the time longed to have a song composed to one of their poems.

## Modern poetry and onomatopoeia

Let us now look at modern poets in this context. The movement to create a new form of Japanese poetry was also a quest for the prose poem, discarding or reorganizing the traditional 7-5 syllable meter. The reorganization of the 7-5 syllable meter was practiced by the early translators of European poems. Here is the first stanza of Paul Verlaine’s poem, “Chanson d’automne (Autumn’s Song, 1867)” and the Japanese version translated by Bin Ueda in 1905:

            Les sanglots longs                                           Aki no hi no

            Des violons                                                      Violon no

            De l’automne                                                   Tameiki no

            Blessent mon cœur                                          Mi ni shimite

            D’une langueur                                                Hitaburu ni

            Monotone.                                                       Uraganashi. ( Verlaine,1962: 72, Ueda, 1978: 75)

Although Ueda’s version is an adaptation rather than a translation, the rhythm of the poem is vivid in Japanese, using the repetition of a five syllabic meter. This version has been appreciated as a perfect example of “excellent translation” in the 20 ^th^ century, with Donald Keene remarking: “But how much superior his Japanese version is to the English one!” ( Keene, 1984: 227).

As a poet of keen senses, Hakushū Kitahara also applied his ingenuity to develop the sound and rhythm of his poems, adopting the meter of *Imayō* and *Kouta*. His first poem put to music was “ *Sora ni Makka na* (In the Sky, Deep Red)” from his first collection *Jashūmon* (Heretical Faith) published in 1909.

            In the sky, deep red are the clouds.             Sora ni makka na kumo no iro.

            In my glass, deep red is the whiskey,         Hari ni makka na sake no iro.

            Why do I feel so sad?                                  Nande kono mi ga kanashikaro.

            In the sky, deep red are the clouds.             Sora ni makka na kumo no iro.

            (Translated by Margaret Benton Fukasawa, 1993: 36–37, Kitahara, 1984:29)

It is said that the members of “Pan Society” had chorused this poem to the melody of *Rappa Bushi* (Trumpet Tune) that was prevalent in the streets at the time ( Nakamura, 1993: 96). Notably, the first music that Kitahara adopted for his poem was a popular song. The repetition of the beat 3, 4 and 5 in the rhythm of *Imayō*—an ancient verse form consisting of four lines each divided into two parts of seven and five syllables—convinces us that Kitahara was a poet interested in “singing poetry” from the beginning.

Combining poetry and music, he made use of sound-symbolic words. Kitahara tried the artistic expression of sound-symbolic words in *Heretical Faith* and developed them in his later works, as a result of the achievement of musicality in poetry. In the poem “Sake to Tabako ni (With Wine and Cigarette)”, he uses three mimetic words effectively— *uttori* (enchanted), *ukiuki* (happily excited), and *shikushiku* (whimper)—making the best use of the repetition of the 7-5 syllable meter. These mimetic words not only made it easier to keep the 7-5 syllable meter, but also gave the poems a lively rhythm and a sense of visual dynamism through the use of hiragana script. ^3^ Kitahara was a pioneer in using sound-symbolic words in his poems.

However, the translation of sound-symbolic words has always been problematic, as the number and use of sound-symbolic words varies from language to language, and in many cases it is not possible to translate verbatim. Eugene Nida pointed out that in some languages “onomatopoeic expressions are considered equivalent to slang”, whereas they are “not only highly developed, but are regarded as essential and becoming in any type of discourse” ( Nida, 2003: 169). Lafcadio Hearn translated some parody poems from *Kyōka Hyaku Monogatari* (A Parodic Poetry on Japanese Ghosts and Goblins), edited by Rōjin Tenmei (Old Tenmei) and published in 1853, as “Goblin Poetry” in *The Romance of the Milky Way and Other Studies and Story* (1905). He chose one poem with a peculiar onomatopoeia:

             Tsuka-no-ma ni

             Hari we tsutawaru,

             Rokuro-Kubi

             Kéta-kéta warau—

             Kao no kowasa yo!

             Swiftly gliding along the roof-beam (and among the props of the roof), the Rokuro-Kubi laughs with the sound of “Kéta-kéta” —oh! the fearfulness of her face!                                 ( Hearn, 1905: 71)

The laughing sound of the long-neck goblin, Rokuro-Kubi, is not translated. Instead of translating the onomatopoeia, Hearn writes in the footnote: “‘ *Kéta*’ means a cross-beam, but *Kéta-kéta warau* means to chuckle or laugh in a mocking way. Ghosts are said to laugh with the sound of Kéta-kéta”.

The translators of modern Japanese poetry have often followed Hearn’s way. Donald Keene translated Sakutarō Hagiwara’s “Neko (Cats, 1917)” as follows:

            Two jet-black cats

            On a melancholy night roof:

            From the tips of their taut tails

            A threadlike crescent moon hangs hazily.

            “ *Owaa*, good evening.”

          “ *Owaa*, good evening.”

          “ *Owaaa*, the master of this house is sick.” ( Keene, 1984: 268–269)

Here, two cats meowing is the transliteration of the Japanese sound. Although Keene points out that “Hagiwara experimented with the musical values of the colloquial and of onomatopoeia” ( Keene, 1984:267, he does not mention that this “Owaa” is not a common Japanese onomatopoeia for a cat meowing. Moreover, he misses the second last sentence of the poem, “ *Ogyaa, ogyaa, ogyaa*,” another strange sound for a cat. ^4^ It is questionable if English readers understand the quality of this poem. Although translators’ efforts have always been enormous, experimental uses of sound-symbolic words by modern Japanese poets have often been untranslatable, making the translations incomprehensible or dull. Here is another example:

          Circus (translated by Noriko Thunman)

          There were several eras

          There was a brown war

          There were several eras

          In winter the gales blew

          There were several eras

          A brief flourishing—here, tonight

          A brief flourishing—here, tonight

          The high rafters of the circus tent

                There, one swing

          Not one you’d notice, a swing

          With head upside down and hands hanging

                Below the dirty cotton roof

          Yuaan, Yuyoon, Yuyayuyon.

          And nearby, a white flame

                 Exhaling sharply like a cheap ribbon

          The spectators are all sardines

                 Their throats whistle like the shells of oysters

          Yuaan, Yuyoon, Yuyayuyon

                  Outside it’s pitch black, the darkness of darkness

                  The long, long night deepens and deepens

                  Along with the nostalgia of the guy

                  with the parachute and

                  Yuaan, Yuyoon, Yuyayuyon ( Thunman, 1983: 75–76)

Chūya Nakahara’s poem, “Circus”, was written in 1929 and published in his anthology *Yagi no uta* (Goat Songs, 1934). In addition to the poet’s particular attention to sentence layout (the translation follows the arrangement of the original), this poem is well-known for its experimental use of the phenomime “ *Yuaan Yuyoon Yuyayuyon*”, a word coined by the poet which mimics the swinging trapeze. Tōru Kitagawa points out the importance of this mimetic word:

The image of the trapeze, swaying back and forth with the onomatopoeia “ *yuaan, yuyoon, yuyayuyon”* under the dirty canvas of the tent, symbolizes the upset or crisis of Nakahara’s obstructed self-consciousness. This intense onomatopoeia moves in response to the swaying of an insecure feeling, rather than arousing the nostalgia. ( Kitagawa, 1968:86)

It should be noted that the typography of the sound-symbolic words is also important in this case. Using the hiragana script **ゆあーん　ゆよーん　ゆやゆよん**, the poet depicts the stretching body of thee flying trapeze performer. It is not only the sound that matters here: the script type, the shape, and the visual effect are also very important. Unfortunately, the transliteration is not able to convey this information. The Roman transcription “ *yuaan, yuyoon, yuyayuyon*” might be seen as a strange enumeration of sound. Consequently, another translator, Christian Nagle, adopted a more target-oriented sound-symbolic wording in a 2013 version: “ *see saw see and saw*”. 

          Circus (translated by Christian Nagle)

          for a number of eras

               there was a brown war

          for a number of eras

          a gale blew in winter

          for a number of eras

          here tonight a drinking party

          here tonight a drinking party

          there is a high beam in the circus tent

               just one trapeze

          an invisible trapeze

          under soiled canvas of the big top

            head down arms dangling

*see saw see and saw*

          close to which white light

                exhales the words “cheap ribbon”

          the audience are a bunch of sardines

          throats gurgle oyster shells

*see saw see and saw*

          outside is dark dark on dark

          night waning forever

          with nostalgia for the damned parachutes

*see saw see and saw*                       ( Nakahara, 2013)

## The eloquence of sound-symbolic words in manga

Perhaps the genre that best utilizes the visual appearance of sound-symbolic words in texts is comics. Onomatopoeia and mimetic words have always been a significant element of comics and graphic novels, because we read them as sound effects. As *Ka-Boom!: A Dictionary of Comic Book Words, Symbols & Onomatopoeia* ( Taylor, 2007) shows, the comics genre has coined many new sound-symbolic words.

Japanese comics, or manga, have particularly developed the artistic expression of sound-symbolic words. Usually placed outside speech balloons, these words are depicted elaborately though varying shapes, sizes, and texture. Manga artists have always tried to figure out ways of expressing onomatopoeia and mimetic words; a phenomime which represents “silence” was even invented in the 1950s ( Tezuka, 1977: 108). Fusanosuke Natsume coined a word *on’yu* for the rich sound-symbolic words in manga, and states as follows:

As a result of diverse inventions and conversions, the onomatopoeia and its group in the manga actually even exceed the category of onomatopoeia— *phonomimes/phenomimes/psychomimes*— contributing to the “multi-layering” and “differentiation” of manga’s vocabulary. ( Natsume, 1995:127)

**Figure 1.**
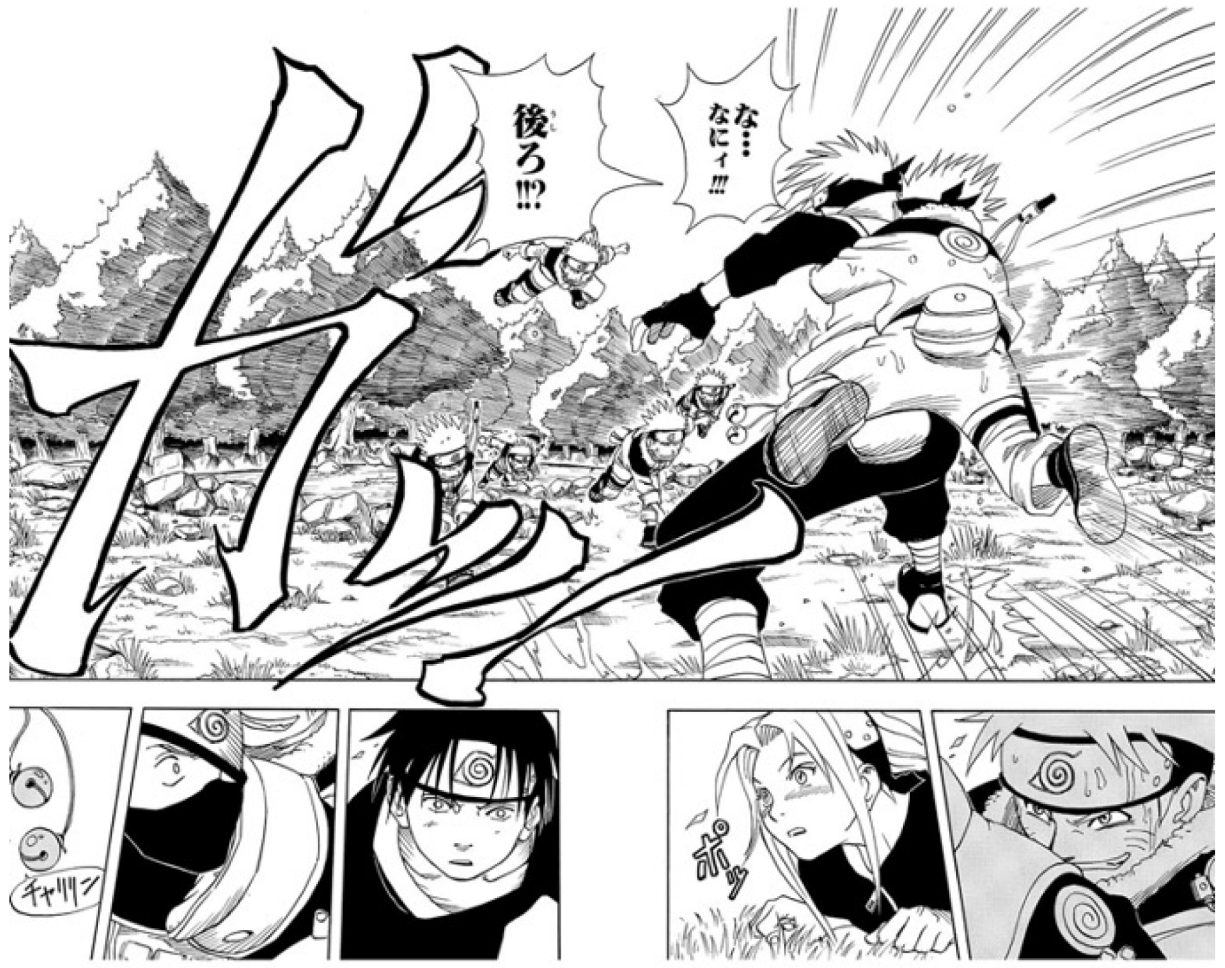
Masashi Kishimoto, *Naruto*, vol. 1 ( 2000). ©2000 Masashi Kishimoto/Shūeisha. This figure has been reproduced with permission from Shueisha.

Sound-symbolic words are considered one of the distinguishing features of manga. Figure 1 is from *Naruto*, Masashi Kishimoto’s blockbuster work. In this scene, the protagonist (Naruto) succeeds in his mission to ring the bell during a training session at the Ninja School, catching the instructor from behind. It is notable that the biggest space in this two-page spread panel is spared for the onomatopoeia *ga!,* the grabbing sound. Hinata (1986: 57) compares onomatopoeia in manga to sound effects in movies. Fukuma (1993: 190) points out that the “the size and font of hand-written onomatopoeia visually explain the volume of a sound, or the speed of an action”. The strong presence of sound-symbolic words in the panel expresses the energy of this scene.

Although hiragana and katakana have been the main writing devices used for the sound-symbolic words in manga, the modern English alphabet has also been used. While the use of Roman letters is not common in major Japanese manga compared to the cases in other countries, discussed by Valero Garcés (2008), we can find the ingenuity of some Japanese artists in words such as “BOMB!” (the sound of an explosion in English) in Akira Toriyama’s Dr. *Slump* ( 1981), or “FLOAT,” “BOOOM,” and “BOOO” in Kōhei Horikoshi’s *Boku no Hero Academia* ( My Hero Academia, 2015). Furthermore, the sound-symbolic words in manga have created characters that were not originally possible. Yōji Yamaguchi points out the expressions of vowels with two dots (voicing mark) are popular in manga ( Yamaguchi, 2014).

Notably, the global popularity of the genre by digital diffusion ^5^ developed a new phase of intercultural dialogues surrounding these words. It is easy to imagine that the translation of sound-symbolic words in manga can pose problems. However, various ways of translating have emerged, depending on target language and/or culture, or the policy of the publishers ( Jüngst, 2008; Schodt, 2016: 7; Sell, 2011: 99–101). This paper hypothesizes that there are three ways thereof:

1. Translation of all sound-symbolic words

2. Preservation of the original expression and putting translation aside

3. Leaving Japanese original sound-symbolic words untranslated, without any explanation

Among the collected examples analyzed for this paper, the first method seems to be the most common in English translations. French translations tend to adopt the second method, ^6^ while the third method seems to apply with Chinese translations. However, further analysis and discussion is needed. ^7^

 Early examples from the last decade of the twentieth century show translators had found a way to translate phonomimes by changing their size, shape, font, and color. They often coined words as well. Heike E. Jüngst observes an intriguing case in German translation of Sadamoto and Gainax’s *Neon Genesis Evangelion* (1999) and states: “The use of onomatopoeia in translation can be very creative” ( Jüngst, 2008). The translation of phenomimes was more difficult. Translators belabored to devise methods, as they sometimes could not find an equivalent word in the target language. James Rampant conducted an analysis of the English translation of Rumiko Takahashi’s *Ranma 1/2* published in 1993 by Viz Media, where the pages were mirrored to change the reading direction from right-to-left into left-to-right, and the translators (Gerald Jones and Matt Thorn were credited for “adaptation”) sometimes changed or omitted the mimetic words. He pointed out that some phenomimes “have been translated with completely new dialogue, *expansion*, which is an example of the adaptation process that takes place in the production of the translation” ( Rampant, 2010: 225). 

For psychomimes, explanations are often added instead of translated. Previous studies have clarified that *shōjo* manga (a manga subgenre for girls) have made “discoveries of the inner self” ( Otsuka, 1994) and have used multiple layers of language for elaborate psychological descriptions ( Natsume, 1995; Otsuka, 1994; Yoshimoto, 2009). We can also find all the elaborated psychomimes to explain the characters’ feelings in this genre. In Karuho Shiina’s *Kimi ni Todoke* volume 1 (Hope it Reaches You, 2006), the emotional chemistry of the silent heroine is often told through phenomimes and psychomimes. As she is not good at expressing her emotions, Sawako, the heroine, often stares at someone with a blank expression or is moved by someone in secret. *Ji,* the phenomime for her stare, and *jiin*, the psychomime for being moved, are used repeatedly ( Shiina, 2006). In the English version *Kimi ni Todoke: From Me to You* (2009), *ji* turns to STARE ( Shiina, 2009: 14, 40, 129, 130, 131), and *jiin* is changed to SO HAPPY (ibid: 17) or to OVERWHELMED (ibid.: 17, 77, 120, 122, 124, 156, 165). In the French version *Sawako* (2010), *ji* is translated to REGARD FIXE (fixed gaze, Shiina, 2010: 14, 40, 129, 130, 131), while *jiin* turns to ÉMOTION (ibid.:17, 77) for the first two cases and ÉMUE (moved) for the rest (ibid.: 120, 122, 124, 156,165).

It is also worth noting here the cases of transliteration. ^8^ In the French version of Gōshō Aoyama’s *Meitantei Conan* (Detective Conan; English version title is *Case Closed*) volume 61 (2010), many sound-symbolic words are transliterated: *ban*, a banging on the desk turns to BAM ( Aoyama, 2010: 7); *bin*, the sound of stretching a string is VIM (ibid.: 17); a rupture tone *pon* is POM (ibid.: 27). The sound of a door opening is expressed in several different ways. Translator Misato Raillard translates the bigger sound *gacha* to CLAC and the smaller sound *cha* to TCHAC (ibid.: 123). The transliteration of the sound of a house burning in a fire, *goooo* (ibid.: 80) and *dong* (ibid.: 96), the sound of heavy objects being put down, is also interesting. Although these transliterations are not always adopted, we can find similar cases in other works. ^9^ Moreover, French translations adopt the strategy to fit both the Japanese original text and the translation in the same panel. Japanese language learners may find this juxtaposition appealing.

## Challenges of global comics: The emergence of hybrid sound-symbolic words

This combination of Japanese writings (especially hiragana and katakana), sound, and the target language seems to influence the visual comprehension of sound effects among readers worldwide. The global influence of Japanese manga has grown considerably in the last decades of the 20 ^th^ century. There are currently many manga-styled comic works in various countries, ^10^ both in the form of magazines and books. In these works, we find some remarkable representations of sound-symbolic words, and some use Japanese sound-symbolic words. For example, Luca Molinaro and Giorgio Battisti juxtapose Japanese and Italian sound-symbolic words in their original Italian manga ^11^ ( Molinaro & Battisti, 2018). Loanwords and their derivations are also impressive. Sell (2011) pointed out the use of OHOHO, a feminine haughty laugh common in Japanese fiction, in *Hollow Field* (2007–2009), a work created by an Australian artist, Madeline Rosca ( Sell, 2011: 99–100). Let us explore these cases in more detail. *Odd Thomas* is a thriller novel series written by Dean Koontz, first published in the United States in 2003. The series is about a 21-year-old short-order cook named Odd Thomas, who has the power to see the lingering dead. Following the success of the novels, three graphic novels and a movie have been released. The first graphic novel, *In Odd We Trust* (2008) was a collaborative work of Koontz and Queenie Chan, an Australian based manga-style comic book artist. In this work, we see some sound-symbolic words in Japanese: PU, to show Odd is blowing out ( Koontz & Chan, 2008: 7, Figure 2), and SHU, the sound of cooking (ibid.: 12, Figure 3). Another noteworthy aspect of the use of these onomatopoeias is the way they are drawn. The addition of prolonged sound marks, which indicates a long vowel of two morae in length in Japanese, and the vertical writing are characteristic of the manga style. It is important to note that these expressions are acceptable to readers of the *Odd* series, who may not be particularly fans of Japanese manga.

**Figure 2.**
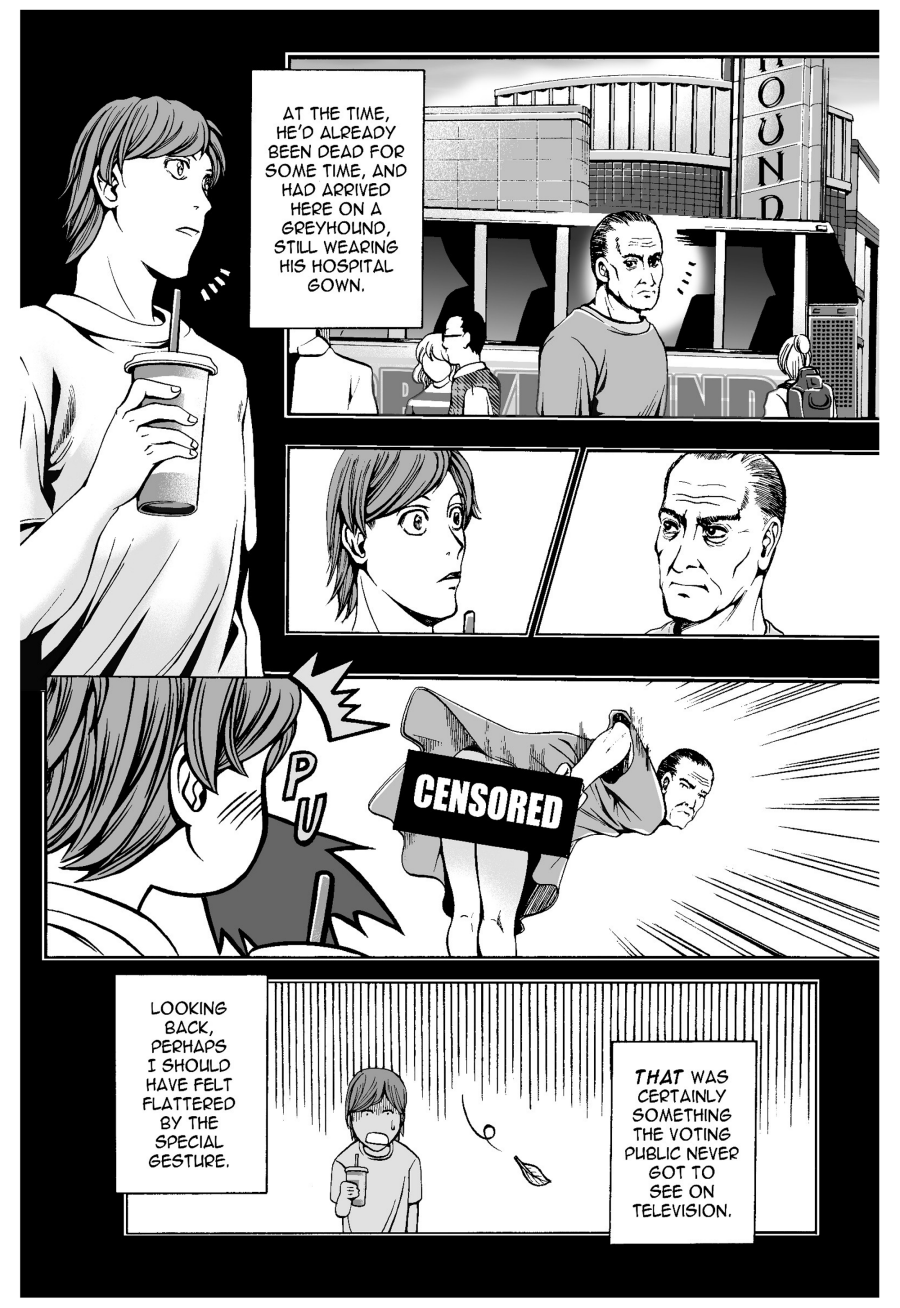
Dean Koontz and Queenie Chan, *In Odd We Trust* (2008) p.7. Illustration © 2008 Queenie Chan / Del Rey Books. These figures have been reproduced with permission from Queenie Chan.

**Figure 3.**
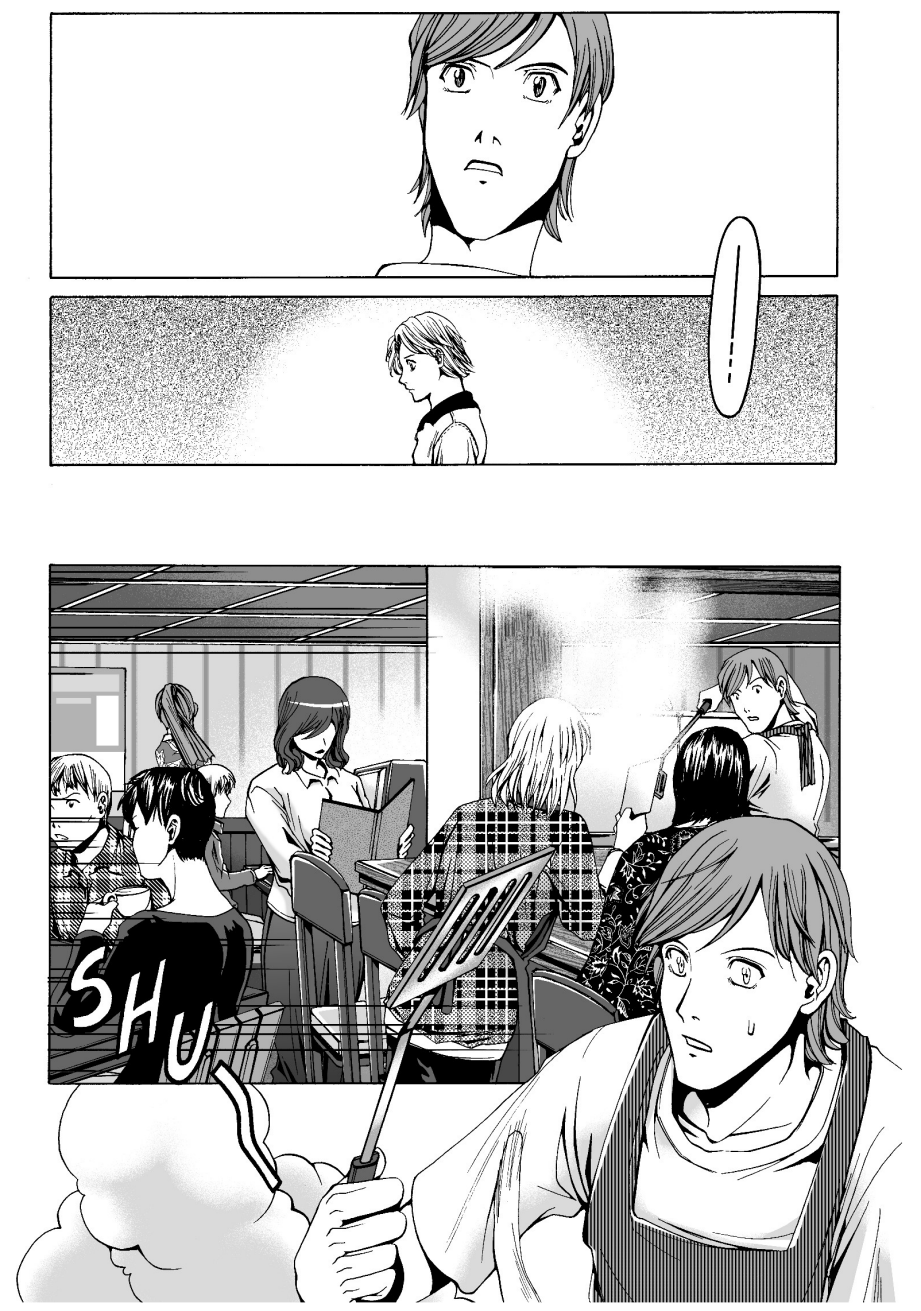
Dean Koontz and Queenie Chan, *In Odd We Trust* (2008) p.12. Illustration © 2008 Queenie Chan / Del Rey Books. These figures have been reproduced with permission from Queenie Chan.

Jenny’s *Pink Diary* was first published in 2005 as *shōjo*-manga styled BD in France, with the story set in Japan. The series won the 14 ^th^ Anime & Manga Grand Prix of *Animeland* magazine, in the category “Best manga-styled BD” ( Olivier, 2007). In volume 2 of this series, the sound of the classroom door opening is represented as CTHAC! ( Jenny, 2006: 8, 15). This could be a derivative onomatopoeia of TCHAC as observed earlier. The volume shows another notable onomatopoeia: KIIYAAH (ibid.: 41, 72, 100) for a women’s scream, which reminds us of *Kyaa,* a common phonomime for women’s scream in Japanese. These examples anticipate the emergence of hybrid sound-symbolic words in the genre.

This hybridity of sound-symbolic words is also notable in Indonesia. Indonesia has a long history of comic works, and the influence of Japanese anime and manga on these works has grown in the late 20 ^th^ century ( Surajaya, 2010: 245). Manga-styled comic works in particular have developed in the country, with several magazines publishing original manga regularly.

*Kyaa*, the phonomime for a girl’s scream which was used in the aforementioned *Pink Diary*, is often seen in Indonesian comics in the form of KYAA, or KYAAA ( Fauziyyah & Kurniawan, 2017: 63; Kartika, 2016: 50; Nisfihani, 2015: 42). Furthermore, we can also find a different usage of *kyaa*, as a phenomime expressing a woman being happy and talkative, in Indonesia ( Viyanriri, 2017: 123; Zulvikar, 2016: 113).

**Figure 4.**
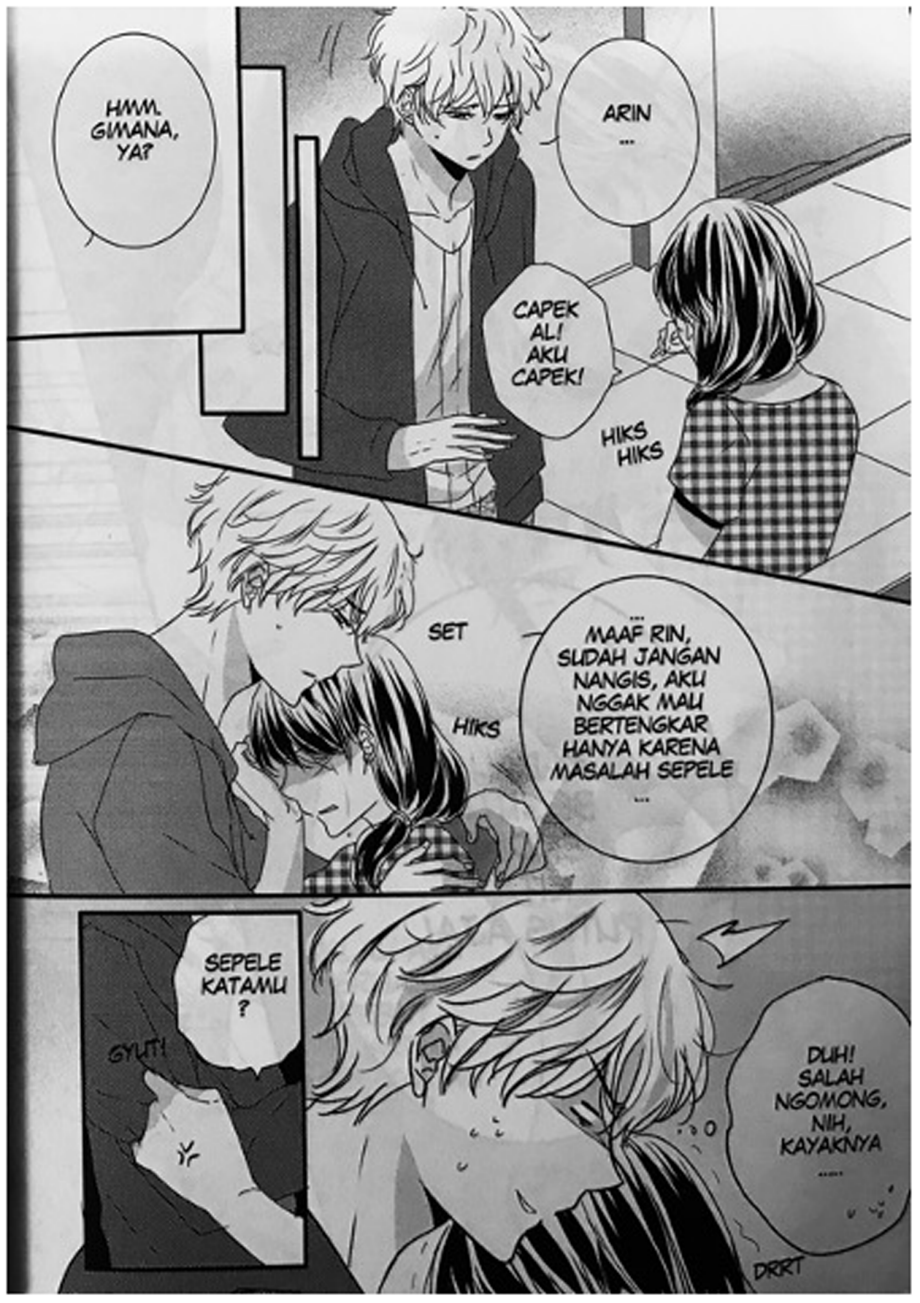
Annisa Nisfihati, *Me vs. Big Slacker Baby,* chapter 6 ( 2016) p. 33. © 2016 Annisa Nisfihani/re:ON Comics. This figure has been reproduced with permission from re:ON Comics.

Another interesting example is the onomatopoeic word HIKS. Annisa Nisfihani is an artist who became popular with her work *Me vs. Big Slacker Baby*, which depicts the delicate sensibilities of teenage girls in *shōjo*-manga style. Nisfihani colors her work with abundant phenomimes and psychomimes which are typical of Japanese girls’ comics. In chapter 6 of this work, published in 2016, we can see in the onomatopoeia HIKS HIKS to show a girl crying ( Figure 4). This seems to be influenced by Japanese onomatopoeia *hick hick*, which usually depicts a child or female crying uncontrollably. What is noteworthy here is that HIKS seems popular in recent manga-styled Indonesian comics ^12^ despite it being non-existent in Indonesian onomatopoeia. Thus, HIKS can be considered a hybrid onomatopoeia emerging from the comic genre in Indonesia.

To understand the common usage of HIKS in this genre, we should consider the influence of the translation. Eriko Ono’s *Kocchi Muite! Miiko* (Look at Me! Miiko, 1995) has become one of the most popular manga for Indonesian girls through its animation series. The Indonesian translation of Ono’s manga was first published in 2002, and its long-lasting popularity led to the artist visiting Jakarta for a “meet and greet” event in 2013. ^13^ The Indonesian version, translated by Widya Anggaraeni Winarya, has some interesting features concerning the sound-symbolic words. Winarya uses some fixed forms of onomatopoeia and mimetic words borrowed from or similar to Japanese, including HIKS. In the two scenes where a girl cries, the onomatopoeia HIKS is used in both cases ( Ono, 2002: 103, 125). However, the original Japanese does not use *hik* in any of the scenes: rather, one scene uses *gusu* ( Ono, 1995: 103), a mimetic word describing crying and sniffling, and the other uses *jiwa* ( Ono, 1995: 125), a phenomime used to describe when tears start to flow. It seems the translator chose HIKS as a fixed onomatopoeia for a crying girl. Another case is the sound of the wind. In this volume, the cold wind in winter is expressed HYUUU ( Ono, 2002: 151, 161, 162, 165). Although *Hyu* is actually a Japanese phenomime for the wind, the sounds *byu* ( Ono, 1995: 151) *, byooo* (Ono, ibid.: 161) *, byuu* (Ono, ibid.: 162, 165) are used in the original. These choices made by translators may have been the foundation for the creation and the diffusion of hybrid sound-symbolic words in Indonesian original comics.

 The hybridization of the appearance of sound-symbolic words is also evident. In Hiro Nurhadi’s *Ankala,* which was serialized in an Indonesian bi-monthly manga magazine, *Shonen Fight*, from 2015 to 2016, ^14^ the artist uses a distinctive font for the sound-symbolic words ( Nurhadi, 2015: 222–245). The font reflects katakana-inspired fonts, such as Tokyosoft created by Shrine of Isis in 1998, or Electroharmonix published by Ray Larabie in 2015. The technique of using stylistic fonts to design pages has also been adopted by French artist Tony Valente, in volume 8 of *Radiant* ( 2017).

## Conclusion

Although Yukio Mishima denounced the use of onomatopoeia as a “childish technique” in the 1950s, modern Japanese texts have persistently developed the utilization of sound-symbolic words. Modern poetry played a leading role in this as poets in the early 20 ^th^ century attempted to create new-style poetry. The pursuit of musicality in modern poetry inspired poets’ artistic use of onomatopoeia and mimetic words. Mixing the scripts and sometimes inventing new sound-symbolic words, they succeeded in giving the new-styled poem a fresh rhythm and visual effect. However, the translation of Japanese sound-symbolic words has always posed a challenge as experimental uses of these words in modern poems were often difficult to translate.

It was graphic narratives and their worldwide distribution that broke through that situation. In this genre, we recognize sound-symbolic words not only as sound effects but also part of the picture, an element inseparable from the story. This recognition invites the reader to take an interest in Japanese sound-symbolic words, and the ingenuity and challenges of the translators are widely appreciated with diffusion through the readership. The parallel notation of Japanese and target languages in the translation of sound-symbolic words deserves special mention. This method has influenced the visual comprehension of phonomime, phenomime, and psychomime among readers worldwide, and created novel expressions; hybrid sound-symbolic words. The cases of emerging hybrid onomatopoeia and mimetic words analyzed and discussed in this paper clarified this process, pointing to the growing importance of sound-symbolic words in the genre, depicted in a variety of ways in comics around the world. They also reveal that the “Third Space” formed by translation and hybridization of manga is indeed a dynamic field that creates a new culture. It is worth examining these words in the genre as they continue to evolve, and we can expect more stimulating examples.

## Data availability

All data underlying the results are available as part of the article and no additional source data are required.

## References

[ref-1] Aoyama G : Detective Conan. translated by Misato Raillard, Bruxelles: Kana.2010;61.

[ref-2] Archie The Redcat: Mulih (Recovery), In *Pulang: Sebuah Kompilasi Komik.*(Home: A Comic Compilation). Jakarta: M&C, Gramedia,2017;5–36.

[ref-3] Bhabha HK : The Location of Culture. Oxon and New York: Routledge.(2004[1994]). Reference Source

[ref-4] Benton Fukasawa M : Kitahara Hakushū: His Life and Poetry. Ithaca: Cornell University Press.1993.

[ref-5] Celotti N : The Translator of Comics as a Semiotic Investigator. In Federico Zanettin (ed.) *Comics in Translation,.*Oxon and New York: Routledge, ebook.2014[2008].

[ref-6] Elisabeth G : My Dear Janitor. In *Permen: Kompilasi Komik Koloni Bergaransi.*(Candy: Compilation of Guaranteed Comics). Jakarta: M&C, Gramedia,2017;55–87.

[ref-7] Fauziyyah A Kurniawan A : The Witness Mirrors. In *Nazo no Kakurenbo.*(Mysterious Hide and Seek), Komik Fantasteen Bandung: Mizan, 2017.2017;49:45–74.

[ref-8] Fukuma Y : Manga ni miru giongo, gitaigo no tokuisei ni tsuite (Peculiarity of Onomatopoeia and Mimetic Words in Manga). *Kyūshu daigaku ryūgakusei center kiyō.* (Research Bulletin of International Student Center, Kyūshu University). Fukuoka: Kyūshu University, 1993;51:85–196.

[ref-9] Haga T : Shiika no mori he (Into the Woods of Poetry). Tokyo: Chūokōron shinsha.2002.

[ref-10] Hara A : Yoshimoto Banana ‘Kitchen’ to Oshima Yumiko ‘Shichigatsu nanoka ni’: ‘Eriko san’ to ‘Kaasama’ (Banana Yoshimoto’s ‘Kitchen’ and Yumiko Oshima’s ‘On July 7 ^th^’: ‘Eriko-san’ and ‘Mother’). *Rekishi bunka shakai ron kōza kiyō.*(Bulletin of History, Culture and Society), Kyoto: Kyoto University.2012;9:53–68.

[ref-11] Hearn L : The Romance of the Milky Ways and Other Studies and Stories. Boston and New York: Houghton Mifflin and Company. Project Gutenberg. Retrieved on June10, 2021.1905. Reference Source

[ref-12] Hinata S : Manga no giongo gitaigo (Onomatopoeia and Mimetic Words in Manga) (2). *Nihon gogaku.*(Japanese Linguistics), Tokyo: Meiji Shoin. 1986;5(8):98–108.

[ref-13] Horikoshi K : Boku no Hero Academia (My Hero Academia). Tokyo: Shūeisha.2015;5.

[ref-14] Iwasaki S : Japanese: Revised Edition. Amsterdam and Philadelphia: John Benjamins Publishing Company.2013. Reference Source

[ref-15] Jenny : Pink Diary. Paris: Delcourt.2006;2. Reference Source

[ref-16] Jüngst HE : Translating Manga. In Federico Zanettin (ed.) *Comics in Translation,.*Oxon and New York: Routledge, ebook.(2014[2008]).

[ref-17] Kartika S : Spalko. chapter 1, In Re *:ON,.*Jakarta: Wahana Inspirasi Nusantara, May 2016.2016;21:37–60.

[ref-18] Keene D : Dawn to the West: Japanese Literature of the Modern Era. (Poetry, Drama, Criticism). New York: Columbia University Press.(1999[1984]);4. Reference Source

[ref-19] Kishimoto M : Naruto. Tokyo: Shūeisha, E-books.(2014[2000]);1.

[ref-20] Kindaichi H : Giongo gitaigo gaisetsu (A Survey of Onomatopoeia and Mimetic Words). In Tsuruko Asano (ed.) *Giongo gitaigo jiten.*(A Dictionary of Onomatopoeia and Mimetic Words) *,.*Tokyo: Kadokawa Shoten.1978.

[ref-21] Kitagawa T : Nakahara Chūya no sekai (The World of Chūya Nakahara). Tokyo: Kinokuniya Shoten.(1971[1968]).

[ref-22] Kitahara H : Hakushū zenshū (Complete Works of Hakushū). Tokyo: Iwanami Shoten.1984;1.

[ref-23] Koontz D Chan Q : In Odd We Trust. New York: Del Rey Books.2008. Reference Source

[ref-24] Maruya S : Bunshō tokuhon (Literary Reader). Tokyo: Chūokōronsha.1977.

[ref-25] Mishima Y : Bunshō tokuhon (Literary Reader). Tokyo: Chūokōronsha.(1995[1959]).

[ref-26] Miyoshi Y : Nihon bungaku no kindai to hankindai.(Modernity and Anti-Modernity in Japanese Literature), Tokyo: University of Tokyo Press,2015[1972]. Reference Source

[ref-27] Molinaro L Battisti G : Death Shield.Brescia: Shockdom,2018;1. Reference Source

[ref-28] Nakahara C : Circus.translated by Christian Nagle.2013; Retrieved on June 10, 2021. Reference Source

[ref-29] Nakamura S : Kitahara Hakushū, Kindai no shijin.(Modern Poets) Tokyo: Ushio Publishing,1993;5.

[ref-30] Natsume F : Manga no Yomikata.(How to Read a Manga) Bessatsu Takarajima EX, Tokyo: Takarajimasha,1995.

[ref-31] Nida EA : Toward a Science of Translating: With Special Reference to Principles and Procedures Involved in Bible Translating.(second edition) Leiden: Brill,2003 [1964]. Reference Source

[ref-32] Nisfihani A : Me vs. Big Slacker Baby.Jakarta: Wahana Inspirasi Nusantara,2015;1. Reference Source

[ref-33] Nisfihani A : Me vs. Big Slacker Baby.chapter 6, In *Re:ON.*Jakarta: Wahana Inspirasi Nusantara,2016;21:9–36.

[ref-34] Nurhadi H : Ankala.chapter 3. *Shonen Fight.* Jakarta: Fajar Waka Semesta,2015;4:222–245.

[ref-35] Olivier : “14e Anime & Manga GRAND PRIX: les vainqueurs (14 ^th^ Anime & Manga GRAND PRIX: the winners).” *Animeland.* 2007; Retrieved on June 10, 2021. Reference Source

[ref-36] Ono M : Nihongo onomatope jiten: Giongo gitaigo 4500.(A Dictionary of Japanese Onomatopoeia: with 4500 phonomimes and phenomimes), Tokyo: Shōgakukan,2007. Reference Source

[ref-37] Ono E : Kocchi muite! Miiko.(Look at Me! Miiko), Tokyo: Shōgakukan,1995;1. Reference Source

[38] Ono E : Hai, Miiko!. translated by Widya Anggaraeni Winarya. Jakarta: M&C, Gramedia,2002;1.

[ref-39] Otsuka E : Sengo Manga no Hyogen Kukan: Kigo teki Shintai no Jubaku.(The Expressive Space of Postwar Manga: The Spell of the Symbolic Body), Kyoto: Hōzokan,1994. Reference Source

[ref-40] Prayoga T Syafrian H : The Story about My Father.In *Permen: Kompilasi Komik Koloni Bergaransi.*(Candy: Compilation of Guaranteed Comics). Jakarta: M&C, Gramedia,2017;151–177.

[ref-41] Rampant J : The Manga Polysystem: What Fans Want, Fans Get. In Toni Johnson-Woods (ed.) *Manga: An Anthology of Global and Cultural Perspectives.*New York: Continuum,2010;221–232. 10.5040/9781628928136.ch-013

[ref-42] Santo I : Shōka to kokugo: Meiji kindaika no sōchi.(Shōka and National Language: A Device for Modernization), Tokyo: Kōdansha.2008. Reference Source

[ref-43] Sell C : Manga Translation and Interculture. In *Mechademia: Second Arc.*Minneapolis: University of Minnesota Press,2011;6:93–108. 10.1353/mec.2011.0002

[ref-44] Shibatani M : The Languages of Japan.Cambridge: Cambridge University Press,2006[1990]. Reference Source

[ref-45] Shiina K : Kimi ni todoke.Tokyo: Shūeisha.2006;1.

[ref-46] Shiina K : Kimi ni Todoke: From Me to You.translated by Tomo Kimura. San Francisco: Viz Media,2009;1. Reference Source

[ref-47] Shiina K : Sawako. translated by Pascale Simon. Bruxelle: Kana.2010.1.

[ref-48] Schodt FL : Translating Manga. In *World Literature Today,.*March/April 2016, Norman: University of Oklahoma,2016;90(2):7.

[ref-49] Simon S : Introduction. In Sherry Simon and Paul St-Pierre (eds) *Changing the Terms: Translating in the Postcolonial Era.*Ottawa: University of Ottawa Press,2000;9–29. Reference Source

[ref-50] Surajaya IK : The Translation of Japanese Manga into Bahasa Indonesia and Manga Mania among Indonesian Youth. In James C. Baxter (ed.) *Globalization, Localization, and Japanese Studies in the Asia-Pacific Region.*Kyoto: International Research Center for Japanese Studies,2010;1:245–257. 10.15055/00001338

[ref-52] Takayama C : Kaitei chūshaku Chogyū zenshū.(Revised and Noted Complete Works of Chogyū), Tokyo: Hakubunkan.1933.7.

[ref-53] Taylor KJ : Ka-Boom!: A Dictionary of Comic Book Words, Symbols & Onomatopoeia.Morrisville: Lulu.com.2007. Reference Source

[ref-54] Tezuka O : Manga no kakikata (How to Draw a Manga).Tokyo: Tezuka Production. (2013[1977]).

[ref-55] Thunman N : Nakahara Chūya and French Symbolism.Stockholm: University of Stockholm.1983. Reference Source

[ref-56] Tokyo University of the Arts and Centennial History Editorial Committee: Tokyo geijyutsu daigaku hyakunen shi. (The Centennial History of Tokyo University of the Arts), Tokyo ongaku gakkō hen (Tokyo College of Music,), Tokyo: Ongaku no tomo sha.1987;1.

[ref-57] Toriyama A : Dr. Slump.Tokyo: Shūeisha. (2012[1981]);5. Reference Source

[ref-58] Ueda B : Teihon Ueda Bin zenshū.(Complete Works of Ueda Bin), Tokushima: Kyōiku Shuppan Center.1978;1.

[ref-59] Valente T : Radiant.Roubaix: Ankama.2017;8. Reference Source

[ref-60] Valero Garcés C : Onomatopoeia and Unarticulated Language in the Translation and Production of Comic Books. In Federico Zanettin (ed.) *Comics in Translation.*Oxon and New York: Routledge, ebook. (2014[2008]).

[ref-100] Verlaine P : *Oeuvres poétiques completes* (Complete poetic works). Paris: Gallimard, 1962.

[ref-61] Viyanriri R : Things Happened.In: *Permen: Kompilasi Komik Koloni Bergaransi.*(Candy: Compilation of Guaranteed Comics). Jakarta: M&C, Gramedia,2017;121–150.

[ref-62] Wolf M : The Third Space in Postcolonial Representation.In in Sherry Simon and Paul St-Pierre (eds) *Changing the Terms: Translating in the Postcolonial Era.*Ottawa: University of Ottawa Press, 2000;127–145. Reference Source

[ref-63] Yamaguchi Y : Tenten: Nihongo kyūkyoku no nazo ni semaru.(Double Dots: The Ultimate Mystery of the Japanese Language), Tokyo: Kadokawa shoten, ebook.2014.

[ref-64] Yoshimoto T : Zen manga ron.(Studies on Manga) Tokyo: Shōgakukan.2009.

[ref-65] Zulvikar F : Wanoja.chapter 7, In *Kosmik Mook.*(Cosmic Mook) Jakarta: M&C, Gramedia,2016;9:105–122.

