# Supplementary material for: Japanese sound-symbolic words in global contexts: from translation to hybridization
Source: F1000Res. 2022 Feb 11;10:1024. Originally published 2021 Oct 8. [Version 2] doi: 10.12688/f1000research.55546.2 (PMC8857522; doi:10.12688/f1000research.55546.2)
Supplement: Peer review history

# Peer review: Reviewer response for version 2

**DOI:** 10.5256/f1000research.121137.r123350

**Published:** 2022-02-18

**Authors:** Rima Devi

**Affiliations:** 1 Universitas Andalas, Padang, Indonesia

This article has been revised to become unified writing following the rules of scientific writing. The conclusion is following the data presented; namely, there has been hybridization in onomatopoeic translation from Japanese manga into the target language. The third space mentioned in this article is described as a hypothesis. The author can use the hypothesis as a research statement for further research.

Is the work clearly and accurately presented and does it cite the current literature?

Yes

If applicable, is the statistical analysis and its interpretation appropriate?

Yes

Are all the source data underlying the results available to ensure full reproducibility?

Partly

Is the study design appropriate and is the work technically sound?

Yes

Are the conclusions drawn adequately supported by the results?

Partly

Are sufficient details of methods and analysis provided to allow replication by others?

Partly

Reviewer Expertise:

Literature, Japanese literature, and Japanese Language Education.

I confirm that I have read this submission and believe that I have an appropriate level of expertise to confirm that it is of an acceptable scientific standard.

# Peer review: Reviewer response for version 2

**DOI:** 10.5256/f1000research.121137.r123349

**Published:** 2022-02-14

**Authors:** Takayuki Yokota-Murakami

**Affiliations:** 1 Graduate School of Language and Culture, Osaka University, Osaka, Japan

The revised manuscript highly successfully mends the shortcomings that the reviewer pointed out. It now connects the first part on the nature of onomatopoeia, the second part on their use in modern Japanese poetry, the third section on the relationship between onomatopoeia and the Western music (as introduced to modern Japan) and the final part treating the significance of onomatopoeia in world comics logically and theoretically. The textual evidence that the author discovered him/herself were also largely added; the initial impression that the paper relies too heavily on preceding researches, giving a few new angles to the phenomenon has now proved to be misled. And the author has given a fully developed conclusion to confer the entire paper theoretical consistency. The manuscript deserves to be indexed.

Is the work clearly and accurately presented and does it cite the current literature?

Yes

If applicable, is the statistical analysis and its interpretation appropriate?

Not applicable

Are all the source data underlying the results available to ensure full reproducibility?

Yes

Is the study design appropriate and is the work technically sound?

Yes

Are the conclusions drawn adequately supported by the results?

Partly

Are sufficient details of methods and analysis provided to allow replication by others?

Partly

Reviewer Expertise:

Comparative literature; Japanese popular culture

I confirm that I have read this submission and believe that I have an appropriate level of expertise to confirm that it is of an acceptable scientific standard.

# Peer review: Reviewer response for version 1

**DOI:** 10.5256/f1000research.59131.r96578

**Published:** 2021-11-17

**Authors:** Rima Devi

**Affiliations:** 1 Universitas Andalas, Padang, Indonesia

In general, this article is interesting because it explains how the translator attempts to translate Japanese into the target language. The translation conveys messages from the source language to the target language: "Communicative translation is social, concentrates on the message and the main force of the text, tends to under-translate, to be simple, clear and brief, and is always written in a natural and resourceful style" (Newmark, 1988: 47-48). There will always be words from the source language that have no equivalent in the target language. Translators try to convey messages from the target language even by removing, adding, or creating new vocabulary in the target language. This translation process makes translators of literary works considered as creators of new works in the target language. In translating poetry and song lyrics, the creation of new words is considered normal because the authors of poetry and song lyrics have been accepted in society as a group that violates standard grammar rules. It can be seen in the poems of Shakespeare (English poet) and Khairil Anwar (Indonesian poet).

 With the creation of new vocabulary by translators when translating the manga into Indonesian, for example, it cannot simply be said that there has been hybridization in translation. New vocabulary is created because there are no equivalent words for onomatopoeic and mimetic words in Indonesian. Meanwhile, in Japanese, there are many onomatopoeic and mimetic words.

 The hybridization proposed by Bhabha (2004) occurred because of colonialization, and the colonized were marginalized and had no place to actualize themselves. Hybridity refers to the creation of new transcultural that exist within the confluence area resulting from colonization: "One of the most widely employed and most disputed terms in postcolonial theory, hybridity commonly refers to the creation of new transcultural forms within the contact zone produced by colonization. As used in horticulture, the term refers to the cross-breeding of two species by grafting or cross-pollination to form a third, ‘hybrid’ species" (Ashcroft et al., 2013: 135-136). Meanwhile, in the translation from Japanese to Indonesian, has colonialization caused this translation? This matter needs further discussion.

 It is too early to conclude that there has been hybridity in manga translation. More accurate data are needed to prove whether hybridization has occurred, and there needs to be adequate evidence and analysis that the third space is a means to publish translated works such as manga.

Is the work clearly and accurately presented and does it cite the current literature?

Yes

If applicable, is the statistical analysis and its interpretation appropriate?

Yes

Are all the source data underlying the results available to ensure full reproducibility?

Partly

Is the study design appropriate and is the work technically sound?

Yes

Are the conclusions drawn adequately supported by the results?

Partly

Are sufficient details of methods and analysis provided to allow replication by others?

Partly

Reviewer Expertise:

Literature, Japanese literature, and Japanese Language Education.

I confirm that I have read this submission and believe that I have an appropriate level of expertise to confirm that it is of an acceptable scientific standard, however I have significant reservations, as outlined above.

## References

[1] : A textbook of translation. *Prentice-Hall International* .1988;

[2] : Postcolonial studies: The key concepts (Third edition). *Routledge, Oxford* .2013;

# Response

**Published:** 2022-02-09

**Authors:** Noriko Hiraishi

**Affiliations:** University of Tsukuba, Japan

Thank you for your valuable comments. I will send you the revised manuscript.

# Peer review: Reviewer response for version 1

**DOI:** 10.5256/f1000research.59131.r96576

**Published:** 2021-11-10

**Authors:** Takayuki Yokota-Murakami

**Affiliations:** 1 Graduate School of Language and Culture, Osaka University, Osaka, Japan

The paper gives an interesting account of the onomatopoeia in Japanese literature and comics and their significance in the instancing of “the Third Space” in the global world culture (in the case of comics). The author attempts to locate the problems with the contemporary theoretical framework, at the same time giving analyses of many intriguing cases in which onomatopoeia are negotiated and “translated” in the comic media. The paper deserves to be indexed, but for the shortcomings listed below, it needs extensive revision.

 The shortcomings of the paper, to my view, are as follows:

1. The paper consists of three sections: a theoretical introduction to the problems of onomatopoeia in Japanese; their historical trajectory in the Japanese modern poetry (mostly from the Meiji period); and the analyses of “translational” use of onomatopoeia in the translation of Japanese manga in the global market. However, the connection among those sections is quite weak. The author mentions in the conclusion that translation of Japanese manga has demonstrated the creative and translational potential of onomatopoeia which was suggested by the poetic experiments in the modern Japanese literature, but the historical trajectory of such a development is not described, that is to say, the connection between them remains purely theoretical. But as the theoretical analyses of onomatopoeia remains under-developed and largely haphazard (for instance, the distinction among the various kinds of onomatopoeia in the linguistical study in the first section is almost neglected in the third section), readers might question the necessity of the first and the second sections. The concept of “musicality” presented in the second section is not fully fledged either (and the readers wonder why the concept has to be brought out).
2. The third section gives interesting and more or less convincing arguments on the various strategies of translating onomatopoeia in world comics. However, whether they constitute, as the author claims, “the Third Space” (which I might term “translational zone” following Emily Apter ^1^) remains somewhat polemical. One might wonder if the cases given are those of adaptation or influence rather than the emergence of “the Third Space.”
3. The case analyses given in the third section, quite stimulating though they are, are mostly taken from the existing previous researches. The author should present more of their own research results.
4. Finally, occasional grammatical and stylistic errors such as “proceed (promote?) the research” (endnote 7).

Is the work clearly and accurately presented and does it cite the current literature?

Yes

If applicable, is the statistical analysis and its interpretation appropriate?

Not applicable

Are all the source data underlying the results available to ensure full reproducibility?

Yes

Is the study design appropriate and is the work technically sound?

Yes

Are the conclusions drawn adequately supported by the results?

Partly

Are sufficient details of methods and analysis provided to allow replication by others?

Partly

Reviewer Expertise:

Comparative literature; Japanese popular culture

I confirm that I have read this submission and believe that I have an appropriate level of expertise to confirm that it is of an acceptable scientific standard, however I have significant reservations, as outlined above.

## References

[1] : The Translational Zone: A New Comparative Literature. *Princeton: Princeton University Press* .2006;

# Response

**Published:** 2022-02-08

**Authors:** Noriko Hiraishi

**Affiliations:** University of Tsukuba, Japan

Thank you very much for your very useful comments.

 I will keep your comments in mind for future work and will further deepen my research.

 Most of the case studies in the third section are the results of the author's own research, not the results of previous studies. I would appreciate your understanding on this point.
